# Analysis of large deletion mutations induced by abasic site analog in human cells

**DOI:** 10.1186/s41021-018-0110-7

**Published:** 2018-10-24

**Authors:** Tetsuya Suzuki, Yuri Katayama, Yasuo Komatsu, Hiroyuki Kamiya

**Affiliations:** 1 Graduate School of Biomedical and Health Sciences, Hiroshima University, 1-2-3 Kasumi, Minami-ku, Hiroshima, 734-8553 Japan; 2 School of Pharmaceutical Sciences, Hiroshima University, 1-2-3 Kasumi, Minami-ku, Hiroshima, 734-8553 Japan; 3 Bioproduction Research Institute, National Institute of Advanced Industrial Science and Technology (AIST), 2-17-2-1 Tsukisamu-Higashi, Toyohira-ku, Sapporo, 062-8517 Japan

**Keywords:** Abasic site, Abasic site analog, Large deletion

## Abstract

**Background:**

Abasic sites are formed spontaneously and by nucleobase chemical modifications and base excision repair. A chemically stable abasic site analog was site-specifically introduced into replicable plasmid DNAs, which were transfected into human U2OS cells. The amplified DNAs were recovered from the cells and used for the transformation of a bacterial indicator strain.

**Results:**

Large deletion mutations were induced by the analog, in addition to point mutations at the modified site. No apparent sequence homology at the deletion junctions was found.

**Conclusion:**

These results suggested that the large deletions induced by the abasic site analog are formed by homology-independent events.

## Background

Chemical modifications of nucleic acids have severe effects on organisms because they disturb replication, transcription, and translation, resulting in mutagenesis, carcinogenesis, and cell death. Various DNA lesions are formed spontaneously and by mutagens, such as reactive oxygen species and ultraviolet light [1–5]. An abasic (apurinic/apyrimidinic, AP) site is formed by the spontaneous hydrolysis of the *N-*glycosyl bonds of DNA, and this hydrolysis is accelerated by the chemical modifications of bases [6]. Moreover, this DNA lesion is produced as an intermediate of base excision repair when a monofunctional DNA glycosylase, such as uracil DNA glycosylase, acts on a damaged base. Approximately 10,000–50,000 abasic sites are reportedly present per mammalian cell under normal physiological conditions [7, 8]. Thus, the abasic site is one of the important DNA lesions causing mutagenesis/carcinogenesis, due to its abundance and loss-of-base character.

In general, the incorporation of a 2′-deoxyribonucleotide opposite the abasic site and its analogs by mammalian replicative DNA polymerases (pols) is inefficient. For example, in vitro DNA synthesis by human DNA pol ε is severely blocked at the nucleotide preceding an abasic site analog [9]. Instead, translesion synthesis DNA pols are considered to conduct the bypass of the DNA lesion [10]. Due to the lack of a nucleobase, DNA pols “misincorporate” nucleotides opposite the DNA lesion. In living mammalian cells, various point mutations are induced by the abasic site (analog), depending on its opposite bases, flanking sequences, and cell type [11–15].

Previously, large deletion mutations were found when shuttle vectors containing ultraviolet-induced DNA lesions, cyclobutane and 6–4 thymine-thymine dimers, were replicated in simian cells [16]. Since these dimers block nucleotide incorporation by replicative DNA pols as with the abasic site [17], it may also cause large deletion mutations in addition to point mutations. In this study, we focused on deletion mutations upon the transfection of a shuttle plasmid DNA bearing a chemically stable, frequently used tetrahydrofuran analog (THF) of the abasic site. The analog actually caused large deletion mutations in living human cells, without any apparent homology at the deletion junctions.

## Methods

### Materials

The oligodeoxyribonucleotides (ODNs) used for plasmid construction and sequence analysis are listed in Table 1. ODN-1 and ODN-2 were previously synthesized [18, 19]. ODN-3 and ODN-5 containing THF, and ODN-4 containing G instead of THF, were synthesized in this study. These ODNs were chemically phosphorylated on the support and purified by HPLC, as described previously [20]. PCR primers were purchased from Hokkaido System Science (Sapporo, Japan) and Eurofins Genomics (Tokyo, Japan) in purified forms. *Escherichia coli* KS40/pOF105, used as the indicator strain of the *supF* mutants, was provided by Professor Tatsuo Nunoshiba of the International Christian University [21].

**Table 1:** Oligodeoxyribonucleotides used in this study

Name	Sequence (5′- > 3′)^a^
ODN-1	P-CGACTTCGAAGGTTCGAATCC
ODN-2	P-CGACTTCGAAGOTTCGAATCC
ODN-3	P-CGACTTCGAAGFTTCGAATCC
ODN-4	P-TCCGAAAGAATTGAGCGTCAGA
ODN-5	P-TCCGAAAGAATTFAGCGTCAGA
ODN-seq1	GGCGGTGCTACAGAGTTCTT
ODN-seq2	GCACCCAACTGATCTTCAGC

^a^F, O, and P represent THF, G^O^, and the phosphate, respectively

### Plasmid DNA containing THF

The double-stranded shuttle vectors containing THF were prepared from the single-stranded forms of pZ189-T_E107K/D402E (formerly pZ189-107 K/402E) and ODN-3 or ODN-5 by enzymatic reactions catalyzed by T4 DNA pol and T4 DNA ligase, as described [22, 23]. To obtain highly pure DNAs, they were treated with Plasmid-Safe ATP-Dependent DNase (Epicentre, Madison, WI, USA). The DNAs were purified with a PureLink PCR Purification Kit (Thermo Fisher Scientific, Waltham, MA, USA) to remove proteins, ODNs, and mononucleotides before and after the DNase treatment. The plasmid DNAs bearing G or 8-oxo-7,8-dihydroguanine (G^O^) were prepared by the same procedures.

### Transfection of plasmid DNAs and mutant frequency determination

U2OS cells (5.0 × 10^4^ cells) were cultured in Dulbecco’s modified Eagle’s medium, supplemented with 10% fetal bovine serum. After 24 h, 100 ng (29 fmol) of the plasmid DNA bearing THF was transfected with Lipofectamine (Thermo Fisher Scientific), and the cells were cultured for 48 h. The plasmid molecules propagated in the U2OS cells was isolated by the Hirt procedure [24]. The DNA was extensively digested by *Dpn*I to break down the unreplicated plasmid. *E. coli* KS40/pOF105 cells were electroporated with the plasmid, and the *supF* mutant frequency was calculated [21, 25]. The plasmids bearing G and G^O^, instead of THF, were transfected as controls. The sequence of the mutant *supF* plasmid was analyzed, using the ODN-seq1 and ODN-seq2 primers (Table 1).

## Results

### Induction of large deletions by an abasic site analog in the *supF* gene (experiment 1)

First, we incorporated THF into position 122 of the *supF* gene. The mutational properties of other DNA lesions, G^O^ and *O*^6^-methylguanine, in this position have previously been examined [18, 19, 26]. The plasmid bearing the unique THF was prepared by enzyme reactions using ODN-3 (Table 1). The DNA was transfected into human U2OS cells, recovered from the cells, and introduced into *E. coli* KS40/pOF105 cells. The numbers of colonies on the titer plate and the selection plates containing nalidixic acid, streptomycin, and X-gal were counted [21, 25]. Mutations in the *supF* gene confer resistance to the two antibiotics and the *lacZ*^−^ phenotype to *E. coli*. The plasmid DNAs containing G and G^O^, instead of THF, were used as controls.

The numbers of colonies on the titer plates, which semi-quantitatively reflect the amounts of plasmid DNA amplified in the U2OS cells, were comparable for all progeny plasmids, indicating the similar replication efficiencies of the G-, G^O^-, and THF-plasmids and THF removal by DNA repair. The *supF* mutant cells formed white or pale blue colonies on the selection plates. The *supF* mutant frequency was calculated by dividing the numbers of *supF* mutant colonies on the selection plates by those of the total colonies on the titer plates.

**Fig. 1:**
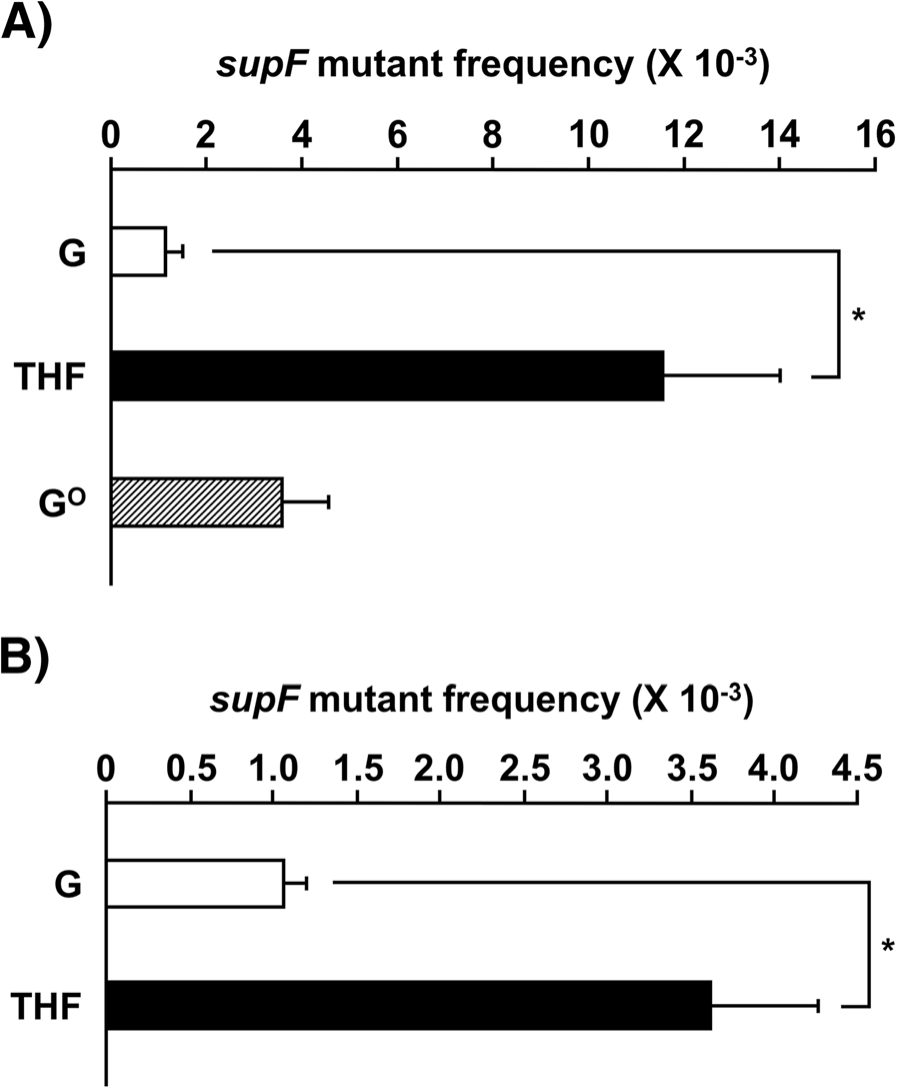
The *supF* mutant frequency in U2OS cells transfected with plasmid DNA containing THF (**a**) at position 122 of the *supF* gene and (**b**) outside of the gene (at “position 171”). Transfection experiments were performed four times. Data are expressed as the means + standard errors. *P < 0.05 vs. control plasmid (Student’s *t*-test)

The *supF* mutant frequency was 1.1 × 10^− 3^ when the control plasmid containing G, instead of THF, was transfected (Fig. 1A). This value was similar to the expected one calculated by the error frequency of the DNA pol used in the plasmid preparation and the length of the *supF* gene, suggesting that a significant portion of the mutations was due to nucleotide misincorporation during the plasmid preparation [27]. The replacement of the G with THF at position 122 greatly enhanced the *supF* mutant frequency (1.2 × 10^− 2^). This value was much higher than the mutant frequency for another major DNA lesion, G^O^.

We next analyzed the *supF* plasmids in the mutant colonies (Tables 2 and 3). Previously, THF has been shown to induce base-substitution mutations [15]. These types of mutations were also found in the experiment. THF➔C and THF➔T mutations were observed as targeted substitutions in this study. In addition, a targeted − 1 deletion was detected. Moreover, as we expected, large deletion mutations were present among the mutants. The frequencies of the large deletion mutations were calculated as the products of the total *supF* mutant frequencies and the ratios of the large deletions. The frequencies were 4.8 × 10^− 4^ and 2.5 × 10^− 3^ in the G and THF experimental groups, respectively. In the case of G^O^, the frequency was 6.4 × 10^− 4^, indicating that not every DNA lesion induces large deletion mutations. Thus, the abasic site analog, but not G^O^, caused large deletions in human cells.

**Table 2:** Mutations detected in the *supF* gene (experiment 1)^a^

THF	
11C- > A, 95C- > A, 101C- > T	1
39G- > GG, 101C- > T, 111C- > T	1
70C- > T, 71C- > T	1
73A- > C, 74G- > A	1
73G- > T, 118G- > A	2
74A- > C, 75G- > A	2
86G- > T, 91G- > A	2
86G- > T, 95C- > A, 101C- > T, 138C- > T, 147C- > T, 161C- > A	1
95C- > G	2
101C- > T, 125C- > G	1
121G- > C, 122G- > T	1
122G- > T	2
large insetion	1
large deletion	6
large deletion, 99A- > G	1
large deletion, large insertion	5
total colonies analyzed	30
G^O^	
5G- > C, 85G- > T	2
5G- > A, 19C- > T, 27G- > A	1
5G- > A, 19C- > T, 27G- > A, 118G- > T	1
5G- > A, 27G- > T, 126G- > C	1
91G- > GG, 118G- > T, 126G- > A	1
95C- > G	1
96 T- > G	2
121G- > T	1
122G- > T	23
122G- > C	8
122 deletion	1
large deletion	7
large deletion, large insertion	1
total colonies analyzed	50
THF	
5G- > C, 27G- > C, 33G- > A, 40G- > A, 73G- > C, 126G- > C	2
5G- > C, 27G- > A, 91G- > T	1
5G- > C, 26C- > A, 27G- > A, 112G- > T, 121G- > T	1
27G- > A, 73G- > A, 118G- > A	1
27G- > C, 65G- > T, 73G- > T	1
27G- > T, 73G- > C, 78G- > T, 118G- > A	1
73G- > C, 106G- > A	1
91 G- > A 112 G- > A	1
91G- > C, 112G- > A	1
91G- > A, 112G- > A, 126G- > C	1
95C- > A, 101C- > T	1
117C- > G, 126G- > T	2
118G- > C	2
121GG- > T	1
122G- > AAGA	1
122G- > T	7
122G- > C	11
122 deletion	11
122G- > T, 127 deletion	1
large deletion	10
large deletion, 122G- > T	1
large deletion, large insetion	1
total colonies analyzed	60

^a^Mutations detected in single colonies are represented. The sequence of the upper strand is shown. The numbers of colonies are shown on the right side

**Table 3:** Spectra of mutations detected in the *supF* gene (experiment 1)^a^

	G	G^O^	THF	
point mutations at position 122				
G:C - > A:T^b^	0 (0)	0 (0)	0 (0)	
G:C - > T:A^c^	3 (10)	23 (46)	9 (15)	
G:C - > C:G^d^	0 (0)	8 (16)	11 (18)	
deletion	0 (0)	1 (2)	11 (18)	
mutations at other positions				
transition				
A:T - > G:C	1 (3)	0 (0)	0 (0)	
G:C - > A:T	16 (53)	8 (16)	17 (28)	
transversion				
A:T - > T:A	0 (0)	0 (0)	0 (0)	
A:T - > C:G	3 (10)	2 (4)	0 (0)	
G:C - > T:A	9 (30)	6 (12)	11 (18)	
G:C - > C:G	4 (13)	4 (8)	19 (32)	
small insertion (1–2 bp)	1 (3)	1 (2)	0 (0)	
large insertion (> 2 bp)	6 (20)	1 (2)	1 (2)	
small deletion (1–2 bp)	0 (0)	0 (0)	1 (2)	
large deletion (> 2 bp)	12 (40)	8 (16)	12 (20)	
others	0 (0)	0 (0)	2 (3)^e^	
total mutations	55	62	94	
total colonies analyzed	30 (100)	50 (100)	60 (100)	

^a^All data are represented as cases found (%)

^b^The mutation corresponds to incorporation of TMP opposite G/G^O^/THF

^c^The mutation corresponds to incorporation of dAMP opposite G/G^O^/THF

^d^The mutation corresponds to incorporation of dGMP opposite G/G^O^/THF

^e^GG - > T at positions 121–122 and G - > AAGA at position 122

### Induction of large deletions including the *supF* gene by an abasic site analog located outside the gene (experiment 2)

Next, we incorporated THF outside the *supF* gene, to detect large deletion mutations more easily. The analog was introduced into the site located 9 “bases” downstream of the gene, which has a length of 162 bases. We named this site “position 171”, although it is outside of the gene. Point mutations at this site alone do not inactivate the *supF* gene, but large deletions containing a part of the gene produce *supF* mutants. ODN-5 was used for plasmid construction (Table 1).

The *supF* mutant frequencies were 1.1 and 3.6 × 10^− 3^ when the plasmid DNAs without and with THF, respectively, were transfected (Fig. 1B). Sequence analyses (Tables 4 and 5) and calculations of the large deletion frequency revealed that THF also induced large deletions in this case: the frequencies were 2.5 × 10^− 4^ and 2.8 × 10^− 3^ for the control and THF plasmid DNAs, respectively (*P* < 0.01, Student’s *t*-test). Thus, we confirmed that the presence of the abasic site analog induces large deletions.

**Table 4:** Mutations detected in the *supF* gene (experiment 2)^a^

G	
5G- > C, 126G- > C	1
65G- > GAA, 71C- > G, 73GAGC- > CCTT, 78GC- > AA, 81A- > G, 85GGA- > ACG	1
70C- > A, 91G- > C	2
70C- > A, 95C- > G	2
91G- > T, 126G- > T	2
95C- > A	2
95C- > G	4
121G- > C	3
122G- > A	1
131C- > A	1
131C- > G	2
large deletion	7
total colonies analyzed	28
THF	
5G- > A, 91G- > C	2
5G- > C, 73G- > C, 112G- > A	1
5G- > C, 27G- > T, 73G- > T, 91G- > T	1
63 deletion	1
73G- > C, 91G- > C	2
95C- > A	1
98A- > T	1
118G- > C	2
large insertion	2
large deletion	33
large deletion, small insertion	8
total colonies analyzed	54

^a^Mutations detected in single colonies are represented. The sequence of the upper strand is shown. The numbers of colonies are shown on the right side

**Table 5:** Spectra of mutations detected in the *supF* gene (experiment 2)^a^

	G	THF
transition		
A:T - > G:C	2 (7)	0 (0)
G:C - > A:T	4 (14)	3 (6)
transversion		
A:T - > T:A	0 (0)	1 (2)
A:T - > C:G	1 (4)	0 (0)
G:C - > T:A	13 (46)	4 (7)
G:C - > C:G	18 (64)	11 (20)
small insertion (1–2 bp)	1 (4)	8 (15)
large insertion (> 2 bp)	0 (0)	2 (4)
small deletion (1–2 bp)	0 (0)	1 (2)
large deletion (> 2 bp)	7 (25)	41 (76)
others	0 (0)	0 (0)
total mutations	46	71
total colonies analyzed	28 (100)	54 (100)

^a^All data are represented as cases found (%)

### Analysis of deleted sequences and junctions

Finally, we analyzed the deleted sequences and junctions. Sequence homology at the junctions of deleted regions should be found, if the deletions occurred in a homology-dependent manner. However, we did not find apparent similarity in any case (Table 6). Thus, homology-mediated events were not involved in the deletion formation.

**Table 6:**
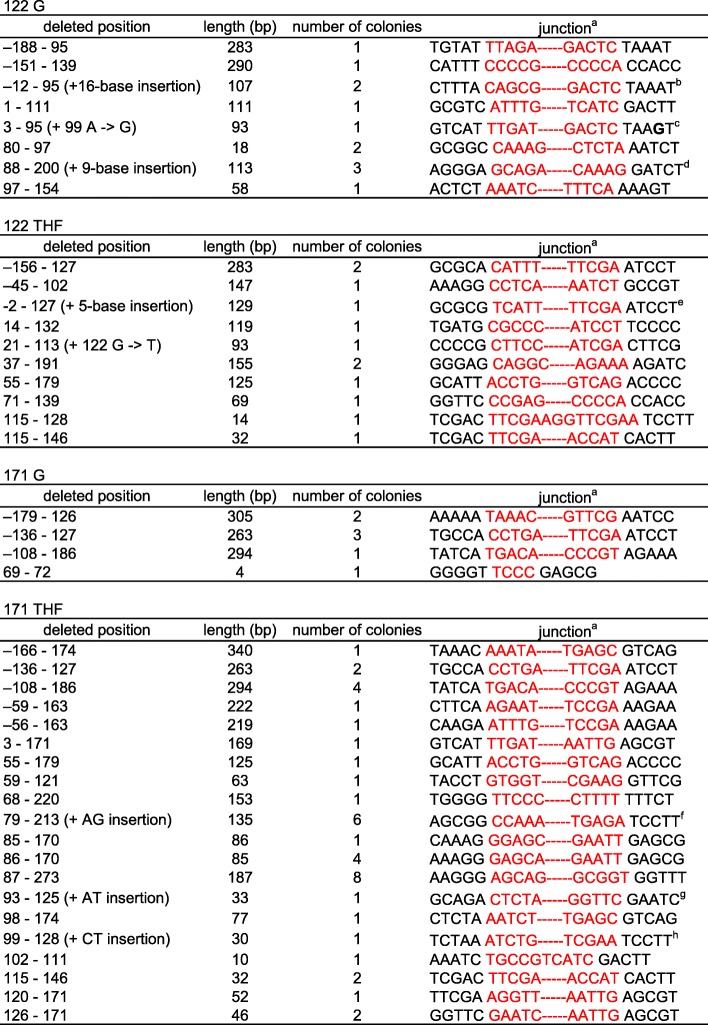
Sequences of deletion junction

^a^The sequence of the upper strand is shown. Deleted portion is shown in red

^b^A TACTGGCCTGCTCCCT sequence is present instead of the deleted sequence

^c^Substitution at position 99 is shown in bold

^d^An ATGCAGCGA sequence is present instead of the deleted sequence

^e^An ATCAT sequence is present instead of the deleted sequence

^f^An AG sequence is present instead of the deleted sequence

^g^An AT sequence is present instead of the deleted sequence

^h^A CT sequence is present instead of the deleted sequence

Twelve mutants obtained in the THF (position 122) experiment contained large deletions. Among them, position 122 was included in the deleted portions for 10 mutants (Table 6). The other two lacked regions upstream of the modified position. No large deletions downstream of the *supF* gene retaining position 122 were found. Meanwhile, position 171 was included in the deleted regions for 26 among the 41 deletion mutants derived from the THF-plasmid (position 171). The other deletion mutants lost the upstream portion of the modified position.

## Discussion

In this study, we examined the large deletion mutations induced by the abasic site analog THF in human cells. Unsurprisingly, substantial fractions of the progenies of THF-bearing vectors contained large deletions (Tables 3 and 5). In the case of THF at position 122 (experiment 1), most of the deleted regions were upstream of the modified site, although the downstream deletion produces *supF* mutants and thus is detectable. Since THF was introduced into the upper strand, the region corresponding to the 5′-side of the lesion was lost. A similar tendency was observed for THF at position 171 (experiment 2), although the lesion was located downstream of the *supF* gene and thus the deletion of the upstream region is essential to inactivate the gene.

When we consider replication, the 5′-side of the lesion is equivalent to the downstream region of the opposite site in the nascent strand. As described in the Introduction section, large deletion mutations were also induced by cyclobutane and 6–4 thymine-thymine dimers in mammalian cells [16]. Since the photodimers and THF are replication blocking lesions, we hypothesize that the inefficient bypass of THF causes the large deletion generated during replication.

Alternatively, the large deletion might be formed as a byproduct of DNA repair. APE1 (APEX, HAP, Ref-1) is the major apurinic/apyrimidinic endonuclease in human cells [28–31]. THF is a substrate of APE1 [32] and the enzyme incises DNA 5′ to THF, resulting in single-strand break (SSB) formation. Op het Veld et al. reported that large deletions were frequently observed in XRCC1-deficient Chinese hamster cells, after a treatment with methyl methanesulfonate [33]. Since the protein is involved in SSB repair and the compound induces the *N*-methylation of nucleobases, they hypothesized that the deletions could be caused by the accumulation of SSBs during abasic site formation and the subsequent incision of the lesions.

Simonelli et al. examined the mutagenic properties of the abasic site in African green monkey COS-7 cells [34]. Although they also found that large deletion mutations (8–168 bases) were induced by the DNA lesion, no information about the deletion junctions was reported. We detected the absence of homology in the deletion junctions we analyzed (Table 6) and concluded that the deletions were due to homology-independent events, such as DNA replication block and/or SSB formation, as described above.

A deaminated base, hypoxanthine, was previously shown to cause an A➔G mutation in mammalian cells [35]. However, DeVito et al. recently reported that the DNA lesion induced large deletions in human HEK293 and HCT116 cells [36]. *N*-methylpurine DNA glycosylase excises this modified base, leaving an abasic site [37]. Since hypoxanthine does not seem to be a replication blocking lesion due to its structural similarity to G, the abasic sites formed by the DNA glycosylase possibly contributed to the deletion mutations, as DeVito et al. discussed. In contrast, G^O^ did not induce large deletions in U2OS cells (experiment 1) although the major repair enzyme OGG1 produces an abasic site. This discrepancy might be due to balance of DNA glycosylase and apurinic/apyrimidinic endonuclease activities. The amounts of the repair proteins would depend on cell types and the abasic sites formed by *N*-methylpurine DNA glycosylase might not be efficiently removed in HEK293 and HCT116 cells. In line with this possibility, G^O^ induces large deletions in HCT116 cells [36]. The repair by monofunctional DNA glycosylases possibly acts as a double-edged sword in cells.

In this study, we found THF➔C and THF➔T mutations at position 122 (Table 3). These substitution mutations seemed to be caused by dGMP and dAMP incorporations, respectively, opposite the lesion. In contrast to *E. coli*, no clear rule of nucleotide incorporation was present in mammalian cells [11, 15]. Interestingly, the − 1 deletion at the THF site (5′-G-THF-T-3′) was observed, in contrast to the previous reports. This mutation might be triggered by dCMP incorporation opposite THF, followed by mispairing of the 5′-flanking G and the incorporated C after looping-out of THF. Alternatively, it might be initiated by dCMP incorporation opposite the 5′-flanking G after looping-out of THF, followed by the extension of the G:C pair.

The detection and quantitation of abasic sites are important research, and useful chemical probes have been developed [38, 39]. Investigations of the mutational properties of abasic sites, as well as their quantitation, are necessary for understanding the contributions of this important DNA lesion to mutagenesis, carcinogenesis, aging, and neurodegeneration.

## Conclusions

In conclusion, the abasic site analog THF induced large deletion mutations in human cells. The lack of homology at their junction sites indicated that the deletions are caused by homology-independent events. Investigations of their molecular mechanism are quite pivotal, and further experiments are currently in progress.

## Abbreviations

G^O^: 8-oxo-7,8-dihydroguanine
ODN: oligodeoxyribonucleotide
pol: polymerase
SSB: single-strand break
THF: tetrahydrofuran analog
